# Valproic acid disables the Nrf2 anti-oxidant response in acute myeloid leukaemia cells enhancing reactive oxygen species-mediated killing

**DOI:** 10.1038/s41416-021-01570-z

**Published:** 2021-10-22

**Authors:** Yao Jiang, Andrew D. Southam, Sandro Trova, Flavio Beke, Bader Alhazmi, Thomas Francis, Anshul Radotra, Alessandro di Maio, Mark T. Drayson, Chris M. Bunce, Farhat L. Khanim

**Affiliations:** 1 School of Biomedical Sciences, Institute of Clinical Sciences, University of Birmingham, Birmingham, UK; 2 School of Biosciences, University of Birmingham, Birmingham, UK; 3 CRUK Cancer Institute, University of Cambridge, Cambridge, UK; 4 Centre for Human & Applied Physiological Sciences, School of Basic & Medical Biosciences, King’s College London, London, UK; 5 University Hospitals Coventry and Warwickshire, Clifford Bridge Rd, Coventry, UK; 6 Institute of Immunology and Immunotherapy, University of Birmingham, Birmingham, UK

**Keywords:** Leukaemia, Drug development

## Abstract

**Background:**

We previously demonstrated the in vitro killing of AML cells by the combination of the lipid-lowering agent bezafibrate (BEZ) and the contraceptive hormone medroxyprogesterone acetate (MPA). A phase II trial demonstrated in vivo safety and efficacy of BEZ and MPA (BaP) in elderly, relapsed/refractory AML and high-risk myelodysplastic syndrome (MDS) patients. However, we observed dose-limiting toxicities in a second trial that attempted to improve outcomes via escalation of BaP doses. Thus we sought to identify a third repurposed drug that potentiates activity of low dose BaP (BaP 0.1 mM).

**Methods and Results:**

We demonstrate that addition of a commonly used anti-epileptic, valproic acid (VAL) to low dose BaP (BaP 0.1 mM)(VBaP) enhanced killing of AML cell lines/primary AML cells to levels similar to high dose BaP (BaP 0.5 mM). Similarly, addition of VAL to BaP 0.1 mM enhanced reactive oxygen species (ROS), lipid peroxidation and inhibition of de novo fatty acid synthesis. Overexpression of Nrf2 in K562 and KG1a completely inhibited ROS production and rescued cells from VAL/BaP 0.1 mM/VBaP killing.

**Conclusions:**

Given the good safety data of low-dose BaP in elderly/relapsed/refractory AML patients, and that VAL alone is well-tolerated, we propose VBaP as a novel therapeutic combination for AML.

## Introduction

Acute myeloid leukaemias (AML) are aggressive blood cancers that, if untreated, kill patients quickly by crippling production of normal blood cells. Treatment is focused on the high rate of cell division in AML cells but this focus also kills the rapidly dividing progenitors of normal blood cells. Consequently, treatment is tempered into short cycles that allow recovery of normal blood cell production from haemopoietic stem cells (HSCs) between treatment cycles. AML is most prevalent in the elderly and >70% of patients are older than 60 years at diagnosis. Many patients, cannot tolerate these repeated cycles of intensive cytotoxic chemotherapy because of their age and disease related frailty [1, 2]. Thus, there is an urgent need to identify therapies that target cancer cell characteristics with no/limited toxicity to normal cells and ideally a good known clinical safety profile.

Drug repurposing (redeployment), where existing drugs are used to treat conditions outside of their approved indications [3], is a proven approach to generate effective low toxicity therapies. This strategy has led to successful blood cancer treatments including all-trans retinoic acid (ATRA) and arsenic trioxide in acute promyelocytic leukaemia [4], and thalidomide in myeloma [5]. We have demonstrated that BaP, the redeployed combination of bezafibrate (BEZ) and medroxyprogesterone acetate (MPA), has in vitro and in vivo anticancer activity in AML, chronic lymphocytic leukaemia (CLL) and non-Hodgkins lymphoma including endemic Burkitts lymphoma [6–11]. Our first clinical trial in AML reported the safety and efficacy of low dose BaP (400 mg per day BEZ and 400 mg per day MPA) in 20 patients (19 AML, 1 high-risk myelodysplasia) for whom chemotherapy was not an option and predicted median survival was 7 weeks (ISRCTN50635541) [10]. Despite being administered continuously rather than in short cycles, no reaction was seen between the drugs and no patient exhibited haematological toxicity from BaP with 11/20 patients taking BaP alone for >4 weeks. One patient reverted from high risk myelodysplastic syndrome (MDS) and remained transfusion independent for >200 weeks of therapy and 3 AML patients gained major improvements in blood cell production for 22–30 weeks; in one, marrow was available to document a partial AML response. This trial used BEZ at lipid-lowering doses that were substantially lower than those that delivered maximal anti-AML activity in vitro [11]. Similarly, MPA was delivered at just under half the optimum in vitro doses. In a subsequent trial in 16 AML patients for whom chemotherapy was not an option (ISRCTN99131400), an increased dose of MPA at 1000 mg per day was well tolerated. However, a twelve fold escalation of BEZ was not well tolerated mainly due to either a rise in Creatinine Kinase (CK) or low estimated glomerular filtration rate (eGFR), resulting in interruptions or changes in BEZ doses [12]. The observed BEZ related toxicity in this trial was due to the frail nature of the patients caused by their age, poor kidney function, disease and in some cases prior chemotherapy treatments. In marked contrast, BaP, at the same high drug doses (adjusted per/kg), was well tolerated in a separate trial in paediatric Burkitts Lymphoma patients (ISRCTN34303497) [8].

Given the success of low dose BaP in our first clinical trial and the problems associated with delivering high dose BEZ in older frail AML patients, we sought to identify an adjunctive low toxicity drug that potentiated the activity of low dose BaP. A screen of our in-house drug repurposing library (FMC) identified the anti-epileptic medication valproic acid (VAL) as a strong candidate. We demonstrate here that low dose BaP, which uses BEZ at 0.1 mM (hereafter referred to as BaP 0.1 mM), when combined with VAL (VBaP) recapitulates selective killing of primary AML blasts and AML cell lines to levels that are similar to high dose BaP (BaP 0.5 mM), whilst not killing primary CD34^+ve^ haemopoietic stem/progenitor cells (HSPCs) or healthy donor blood cells.

We also demonstrate that killing of AML cells by VBaP was associated with enhanced generation of reactive oxygen species (ROS) when compared to BaP 0.1 mM, although not to the levels of BaP 0.5 mM. We demonstrate that VAL treatment of AML cells inhibits the essential Nrf2 antioxidant response pathway by suppressing Nrf2 mRNA and protein expression and activity. Furthermore, induction of ROS and loss of viability by BaP and VBaP was rescued by overexpression of Nrf2. Thus, the triple repurposed drug combination of VBaP works by targeting the inherent sensitivity of AML cell to oxidative stress [13, 14] by inducing ROS whilst attenuating the anti-oxidant response.

## Methods

### Chemicals and reagent

Valproic acid (VAL, 0.6 M in dH_2_O), bezafibrate (BEZ, 0.5 M in DMSO), and medroxyprogesterone acetate (MPA, 5 mM in ethanol) were purchased from Sigma-Aldrich (UK). The active metabolite of vitamin D3, 1,25 dihydroxy vitamin D3 (VD3) and all-trans retinoic acid (ATRA) were purchased from Cambridge Biosciences, and prepared as 1 mM stocks in ethanol. All stocks were stored at −20 °C.

### Cell culture and treatment

KG1a, K562, HL60, NB4 cells were obtained from DSMZ and verified by regular STR profiling. Cell lines were maintained in RPMI 1640 media supplemented with 10% foetal bovine serum (FBS), 100 μg/ml penicillin and 100U/ml streptomycin in a humidified incubator at 37 °C with 5% CO_2_. Primary blood samples from AML patients and normal donors were obtained as surplus to diagnosis samples and from normal donors (REC numbers 12/NW/0742 and ERN_17-0065). Healthy donor blood was obtained after informed consent under University of Birmingham (UoB) local ethical approval ERN_17-0065. Peripheral blood mononuclear cells (PBMCs) were purified by density centrifugation through Ficoll and plated at 1 × 10^6^ cells/ml in RPMI1640 media supplemented with 10% foetal bovine serum (FBS), 100 μg/ml penicillin and 100 U/ml streptomycin.

### Drug treatments

Cells were treated with either solvent control (CON), 0.6 mM Valproic acid (VAL), high dose BaP: 0.5 mM BEZ and 5 μM MPA (BaP 0.5 mM), Low dose BaP: 0.1 mM BEZ and 5 μM MPA (BaP 0.1 mM), or the combination of VAL 0.6 mM and BaP 0.1 mM (VBaP).

### Flow cytometry

All flow cytometry was performed on a BD FACS Calibur utilising CellQuest Pro software and analysed using FlowJo Software.

### Cell viability

Cell viability was determined using one of three assays as specified in the text. Cells were treated in 200 μl in triplicate wells in 96-well plates at 1–2 × 10^4^ cells/well for cell lines and 2 × 10^5^ cells/well for primary cells. Viability was assessed using either: CellTiter-Blue® reagent according to the manufacturer’s instructions (Promega, UK), viable cell counts using manual counts, or by flow cytometry utilising viable gates and fluorescent Cytocount beads (Biolegend, UK). Primary samples were harvested, stained with anti-CD34-APC and anti-CD117-PE (BD Biosciences, UK) before analysis by flow cytometry utilising viable gates and fluorescent Cytocount beads.

### Annexin V and cell cycle analysis

Apoptosis was assessed using an Annexin V-FITC kit (BD Biosciences) according to the manufacturer’s instructions. Cell cycle was analysed by resuspending cell pellets from 500 μl cell suspensions in 500 μl cell cycle buffer (30 μg/ml PI, 0.1 mM sodium chloride, 1% sodium citrate, 1% Triton X100) and incubating at 4 °C for 4 h before flow cytometry analysis.

### Assessment of reactive oxygen species and mitochondrial superoxide (mitosox)

MitoSOX Red (ThermoFisher Scientific, UK) was used to assess mitochondrial superoxide (mitosox) levels. PBS-washed cells were resuspended in 200 μl 37 °C PBS containing 5 μM MitoSOX Red, incubated at 37 °C for 10 min and then analysed by flow cytometry. Reactive oxygen species (ROS) were measured using carboxy-H_2_DCFDA (5-(and-6)-carboxy-2,7-dichlorodihydrofluorescein diacetate, Invitrogen). Cells were treated with solvent control/drugs at 37 °C and 10 μM carboxy-H_2_DCFDA added to 0.5 ml cell suspension for the final 45 min before washing with PBS and flow cytometry.

### GSH/GSSG assay

Reduced glutathione (GSH) and oxidised glutathione (GSSG) were measured with GSH/GSSG-Glo™ Assay Kit (Promega,UK) according to the manufacturer’s instructions. Cells were treated for 4 h before 5 × 10^4^ cells were transferred into 96-well luminescence plates and luminescence recorded using a luminometer (Victor X3, Perkin Elmer Inc., UK).

### Prostaglandin ELISAs

Prostaglandin D_2_-MOX EIA kit (Cayman Chemicals, USA) was used to determine prostaglandin levels. After treating 5 × 10^6^ cells in 5 ml media for 4 h, cells were harvested with 1 ml culture media and homogenised using a Precellys 24 ceramic bead homogenisation system (Bertin instruments, UK). Prostaglandins were extracted using C18 reverse phase extraction columns (Chromabond, Fisher, UK) and levels determined by ELISA according to the manufacturer’s instructions.

### Western blot

5 × 10^6^ treated cells were lysed in RIPA buffer (1% v/v NP40, 0.5% w/v sodium deoxycholate, 0.1% w/v 10% SDS, protease inhibitors) and equal amounts of proteins (20–50 μg) separated on 4–15% gradient SDS-polyacrylamide gels (BIO-RAD Laboratories, USA) before transferring to polyvinylidene fluoride (PVDF) membranes (Millipore Corp, Bedford, USA) and blocking with 5% skimmed milk powder in TBS-T (TBS + 0.1% Tween 20) for 60 min, PVDF membranes were washed three times with TBS/T and incubated with primary antibody at a 1:1000 dilution in TBS/T-5% BSA overnight at 4 °C. After washing with TBS/T, membranes were probed with goat anti-rabbit or anti-mouse IRDye (1:15000) (LI-COR Inc., Germany) at room temperature for 60 min, washed with TBS/T and scanned using the Odyssey Imaging System (LI-COR Inc.).

### Real-time PCR quantification

Reactions were performed using an Applied Biosystem 7000 Systems (Applied Biosystem) as described previously [11] using primers and probes specific to NQO1, GSTA1 and NFEL2 (Nrf2) (Sigma-Genosys, UK and Eurogentec Ltd, Southampton, UK) (Supplementary Methods).

### Confocal microscopy

NB4, KG1a and HL60 cells were treated for 4 h and cytospins prepared before fixing with 4% paraformaldehyde. Slides were stained with an anti-Nrf2 (Abcam Ltd, UK) antibody diluted in PBS + 0.05% Tween-20 (PBS/T) overnight at 4 °C before washing 3x with PBS/T and incubating with secondary antibodies [Alexa Fluor® 594-AffiniPure F(ab’)_2_ Fragment Donkey Anti-Rabbit IgG (H + L) (Jackson ImmunoResearch Europe Ltd., Cambridge, UK) for 2 h at room temperature. After 3 × 5 min washes with PBS/T, slides were mounted with VECTASHIELD® Mounting Media with DAPI (Vector Laboratories Ltd., UK). Confocal microscopy images were acquired with a Nikon A1R confocal system equipped with a 402 nm laser, a multiline Argon laser and a 633 nm laser and 40x (1.3) and a ×100 (1.4) objectives. Images were analysed using ImageJ.

### ^13^C incorporation from ^13^C_6_ D-glucose into free fatty acids

10 × 10^6^ cells at 2.5 × 10^5^ cells/mL were drug- or solvent control-treated for 24 h in either standard media or glucose-free RPMI-1640 (Gibco-Invitrogen) supplemented with 2 g/L ^13^C_6_ D-glucose (Sigma-Aldrich). Cells were harvested by quenching in 60% methanol (at −40 °C), and lipids were extracted using a biphasic chloroform/methanol/water method (solvent volumes normalised to cell biomass) and then dried under nitrogen gas as detailed previously [6]. Dried lipid extracts were resuspended in 2:1 methanol/chloroform with 5 mM ammonium acetate and analysed by direct infusion mass spectrometry (DIMS), using a Triversa nanoelectrospray system (Advion Biosciences; −1.7 kV negative ion mode, 0.3-psi backing pressure) and a hybrid linear ion trap FT-ICR mass spectrometer (LTQ FT Ultra; Thermo Fisher Scientific), in negative ion mode with a resolution of 200,000 (for an ion at 400 *m/z*) as detailed previously [6]. Data were processed and normalised as previously described [6] and then peaks with an *m/z* value matching the accurate masses of free fatty acid C_12_ and C_13_ isotopes (as [M-H]- ionforms; mass error ppm of ±5 ppm) were identified. For a given free fatty acid, data are presented as the total intensity of all its C_13_ isotopes (minus the calculated naturally occurring C_13_ intensity from the C_12_ peak) displayed as mean ± SEM, *N* = 3 [6].

### Stable NRF2 overexpressing cell lines generation

Stable Nrf2 overexpressing AML cells were generated using the two plasmids piggyBac system from VectorBuilder (VectorBuilder Inc., TX, USA) (Supplementary Fig. 1). The transposon plasmid contains a transposable cassette with the hygromycin resistance gene under CMV promoter and the polycistronic NRF2-EGFP genes linked with T2A self-cleaving 2A sequence under the EF1A promoter. EGFP control transposon plasmid was designed identically, but lacking the NRF2 gene and T2A sequence. The transposase plasmid transiently expresses the hyperactive version of piggyBac transposase (PBase) (Supplementary Fig. S1).

5 × 10^6^ K562 and KG1a cells were resuspended in 100 µl of Ingenio Transfection Solution (Mirus Bio LLC, Madison, WI, USA). Highly purified endotoxin free plasmids were added as follows: 1 µg of PBase transposase plasmid and 1.5 µg of either NRF2(T2A)EGFP or EGFP transposon plasmid. Cells were transfected with program U-008 using an Amaxa Nucleofector II (Lonza, Basel, Switzerland), resuspended in 3 ml of warm complete media and incubated for 72 h. K562 and KG1a transfected cells were then put under selection with 200 µg/ml or 300 µg/ml of Hygromycin B Gold (Invivogen, San Diego, CA, USA), respectively. Selection media was changed after 72 h and when needed. After 3 weeks under selection, stably transfected cells were sorted by GFP fluorescence by the University of Birmingham Flow cytometry facility, and polyclonal sorted cells expanded in hygromycin free media.

### Statistical analysis

Unless stated otherwise, data were compared using one-way ANOVA with Tukey post-hoc test. Significance of *p* < 0.05 is denoted by letters. Unique letters highlight statistical difference from the other treatment groups. Treatment groups with the same letter are not significantly different from each other.

## Results

### Valproic acid enhances anti-AML effect of low dose BaP

**Fig. 1:**
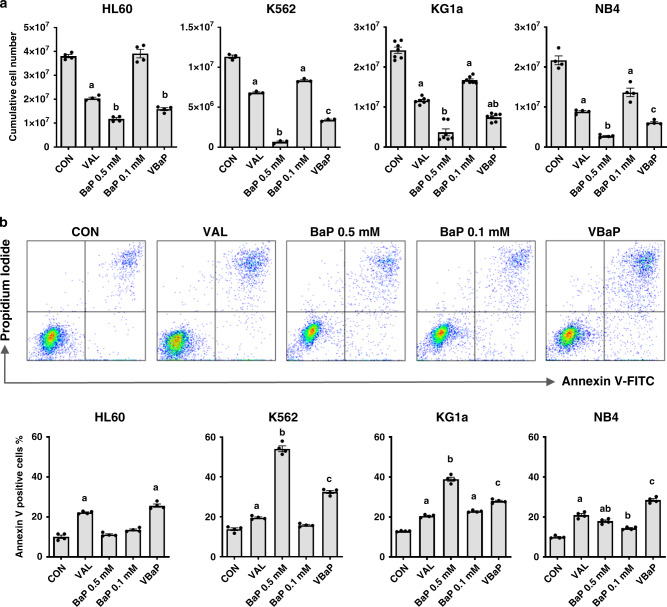
VBaP kills AML cell lines. **a** HL60, K562, KG1a and NB4 cells were treated with vehicle control (CON), 0.6 mM Valproic acid (VAL), 0.5 mM BEZ and 5 μM MPA (BaP 0.5 mM), 0.1 mM BEZ and 5 μM MPA (BaP 0.1 mM), the combination of Valproic acid 0.6 mM and BaP 0.1 mM (VBaP) for 7 days with feeding and retreating every 2 days and numbers of surviving cells determined by flow cytometry. Bar charts shows mean ± SEM for *n* = 3–7 experiments. **b** HL60, K562, KG1a, and NB4 cells were treated as shown for 4 days and Annexin V binding determined using flow cytometry. FACS plots show representative Annexin V/PI staining for NB4 cells and bar graphs show mean ± SEM for *n* = 4 experiments. Different letters indicate significant difference from other treatment groups as determined by ANOVA (*p* < 0.05).

In order to enhance the anti-tumour efficacy of BaP, we screened our custom drug repurposing library (FMC) for an adjunctive agent that increased AML cell killing in the presence of low dose BaP (BaP 0.1 mM). This screen identified sodium valproate (sodium salt valproic acid-VAL) (data not shown) a commonly used oral anti-epileptic agent which is generally very well-tolerated. Fig. 1a shows cell survival data for HL60, K562, KG1a and NB4 AML cell lines treated with vehicle control, VAL, high dose BaP (0.5 mM BaP), BaP 0.1 mM and VBaP (VAL + BaP 0.1 mM). As expected, cell killing by BaP 0.1 mM in all four cell lines was inferior to killing in response to BaP 0.5 mM (Fig. 1a). However, VAL alone and in combination with BaP 0.1 mM (VBaP) enhanced cell killing when compared to BaP 0.1 mM alone at day 7 (Fig. 1a).

VBaP-induced AML cell death was associated with Annexin V (AV) positivity, a marker associated with apoptosis (Fig. 1b). In NB4 and HL60 cells, VBaP induced greater AV positivity than BaP 0.5 mM whereas in K562 and KG1a they were slightly lower. VAL alone increased AV positivity in all cell lines, and in HL60 cells generated higher AV expression than BaP 0.5 mM (Fig. 1b).

**Fig. 2:**
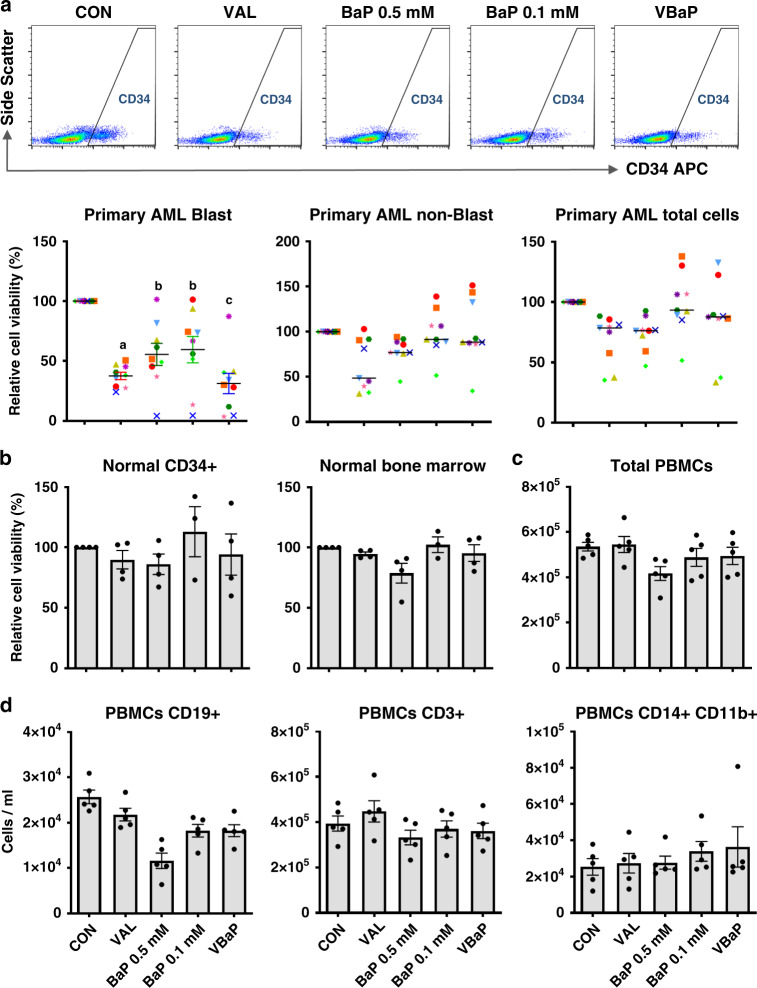
VBaP kill primary AMLs but not normal healthy cells. **a** Primary AML samples were treated for 4 days and the viability of AML blast cells (positive for either CD34 and/or CD117), non-blast CD34^−^/CD117^−^ cells and total cells were determined by flow cytometry. Horizontal bars represent the mean values of all samples. FACS plots show typical gating for CD34^+^ cells. Dot plots show individual sample viable cell counts normalised to each sample untreated control. **b** Normal donor bone marrows (*n* = 4) were treated for 4 days and total CD34^+^ cell number and total cell viability were determined by flow cytometry. (C&D) PBMCs (*n* = 5) were purified from healthy donors and treated for 4 days. Survival of **c** total PBMCs, **d** B lymphocytes (CD19+), T lymphocytes (CD3+) and monocytes (CD14+CD11b+) were determined by flow cytometry with counting beads (*n* = 4). Bar charts shows mean ± SEM. Different letters indicate significant difference from other treatment groups (*p* < 0.05). Abbreviations: vehicle control (CON), 0.6 mM Valproic acid (VAL), 0.5 mM BEZ and 5 μM MPA (BaP 0.5 mM), 0.1 mM BEZ and 5 μM MPA (BaP 0.1 mM), the combination of Valproic acid 0.6 mM and BaP 0.1 mM (VBaP).

Importantly, the combined activity of VBaP compared to either drug alone was not restricted to AML cell lines and was recapitulated against primary AML samples (*N* = 9) (Fig. 2a). In particular, VAL alone and VBaP had increased activity against AML blasts when compared to either BaP 0.5 mM or BaP 0.1 mM (Fig. 2a). In contrast, drug treatments demonstrated variable, but non-significant effects on the non-blast cells in AML samples (Fig. 2a) and this was reflected in total AML cell numbers (Fig. 2a). These data indicate that within AML bone marrow samples, our drugs specifically target the tumour blast cells. This was further supported by data from healthy donor samples. VBaP induced no significant loss in viability in samples from healthy donors including bone marrow derived CD34^+ve^ hematopoietic stem/progenitor cells (HSPC) or total bone marrow cells (Fig. 2b), total peripheral blood mononuclear cell (PBMC) preparations (Fig. 2c), or B cell (CD19+), T-cell (CD3+) or myeloid cell fractions (CD14+CD11b+) within PBMCs (Fig. 2d).

### VBaP reduces SCD1 protein levels and activity

**Fig. 3:**
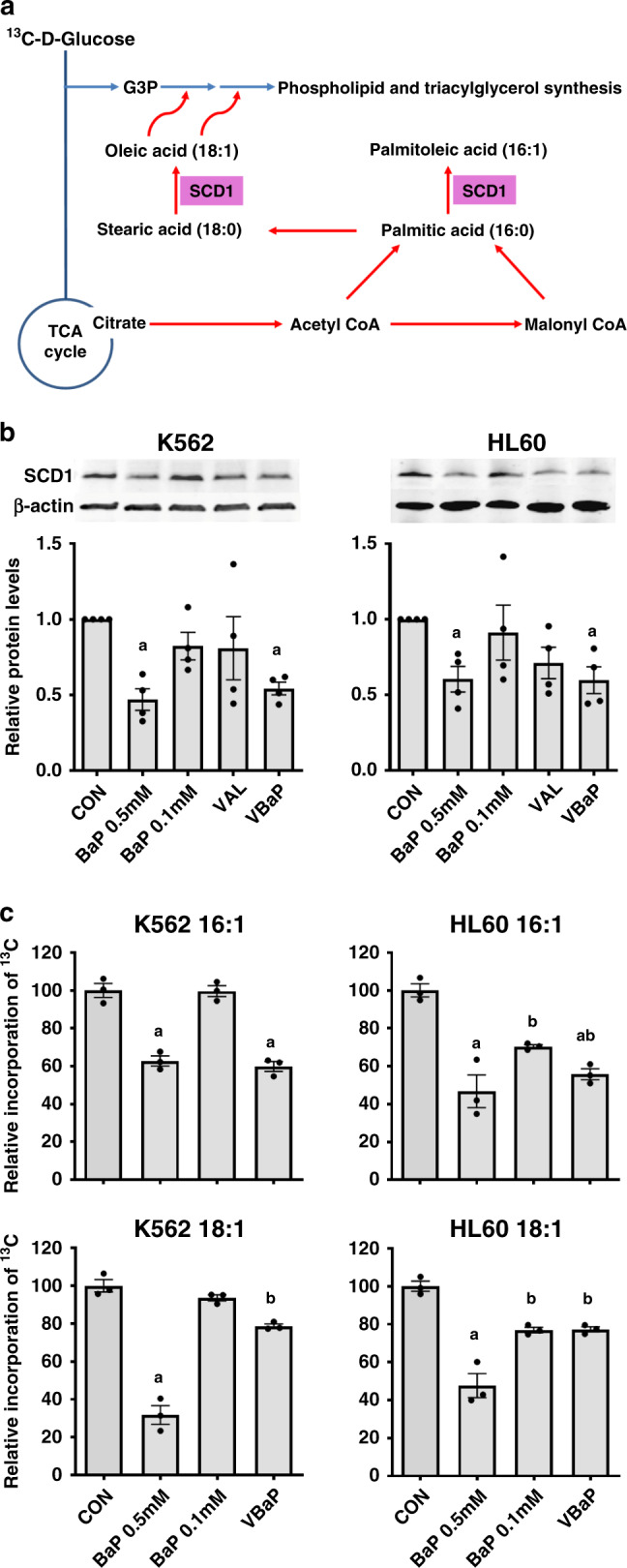
VBaP decreases SCD1 protein levels and activity in AML cell lines. **a** Schema showing role of SCD1 in monounsaturated fatty acid (MUFA) metabolism. **b** K562 and HL60 cells were treated for 72 h and protein levels of SCD1 determined using western blotting. Panels show representative images of western blots. Bar charts show mean ± SEM densitometry data (*n* = 4). **c** K562 and HL60 cells were treated for 24 h and de novo MUFA synthesis from ^13^C-glucose determined using mass spectrometry. Data are shown as ^13^C incorporation into palmitoleic acid (16:1) and oleic acid (18:1) relative to control (*n* = 3, ±SEM). Different letters indicate significant difference from other treatment groups (*p* < 0.05). Abbreviations: vehicle control (CON), 0.6 mM Valproic acid (VAL), 0.5 mM BEZ and 5 μM MPA (BaP 0.5 mM), 0.1 mM BEZ and 5 μM MPA (BaP 0.1 mM), the combination of Valproic acid 0.6 mM and BaP 0.1 mM (VBaP).

Stearoyl-CoA desaturase-1 (SCD1) is a key enzyme in fatty acid metabolism, catalysing the rate-limiting step in the formation of monounsaturated fatty acids (MUFAs), specifically oleic acid (18:1) and palmitoleic acid (16:1) from stearic acid (18:0) and palmitic acid (16:0) (Fig. 3a) [15]. We previously reported that BaP 0.5 mM can disrupt the SCD1-mediated synthesis of MUFAs in AML cells and that this was mediated, in part, by BaP downregulating SCD1 protein levels [6]. BaP 0.5 mM again reduced SCD1 protein levels in K562 and HL60 cells however, BaP 0.1 mM and VAL alone did not (Fig. 3b). In contrast, addition of VAL to BaP 0.1 mM reduced SCD1 protein levels to a similar level as BaP 0.5 mM in both K562 and HL60 (Fig. 3b).

As previously reported [6], mass spectrometry lipidomics analysis after 24 h exposure to ^13^C-glucose identified that BaP 0.5 mM reduced both palmitoleic acid (C16:1) and oleic acid (C18:1) synthesis, an observation consistent with reduced SCD1 levels (Fig. 3c). BaP 0.1 mM had no effect on ^13^C-incorporation into C16:1 and C18:1 in K562, whereas a small reduction was observed in HL60 cells. VBaP treatment drove down C16:1 levels in both cell lines to a level similar to BaP 0.5 mM however, the impact of VBaP on C18:1 levels was less marked (Fig. 3c). All these data demonstrate that at 24 h VBaP targets SCD1 and palmitoleic acid (16:1) levels in AML cells.

### VAL enhanced the ROS generation in AMLs

**Fig. 4:**
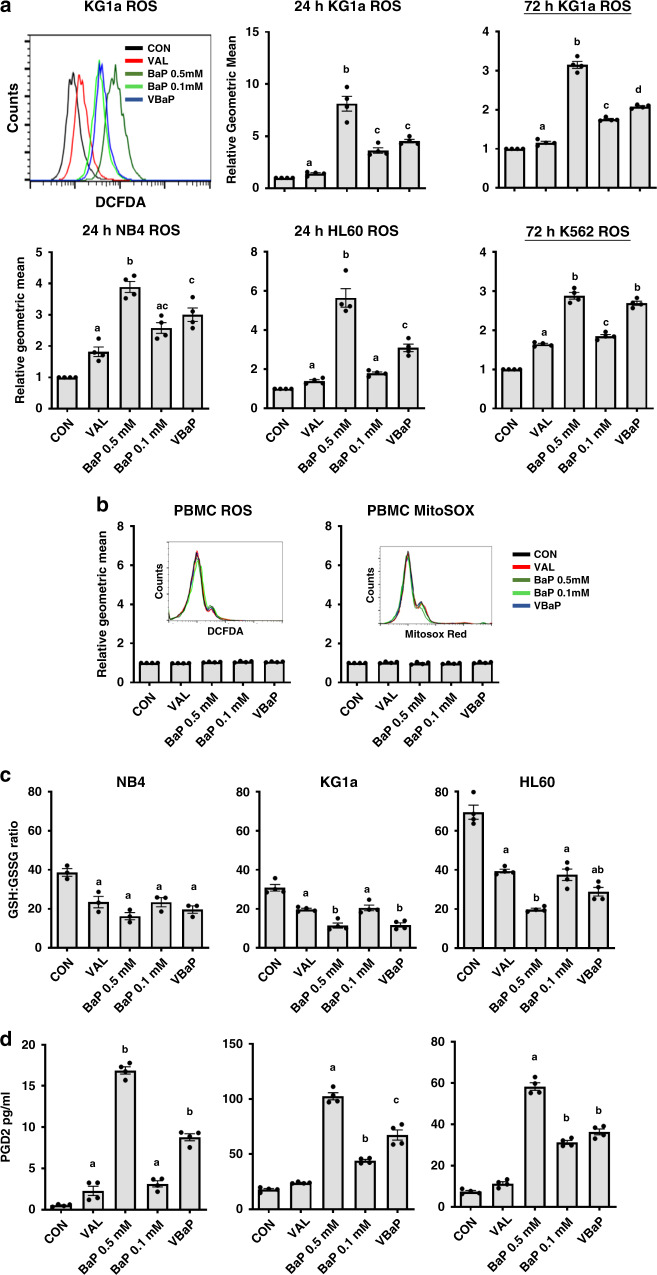
VBaP induced ROS generation in AML cells but not PBMCs. **a** KG1a, NB4, HL60 and K562 cells were treated for 24 or 72 h, stained with carboxy-H_2_DCFDA for the detection of ROS and analysed by flow cytometry. Flow cytometry histograms show representative ROS data after 24 h treatment for KG1a cells; bar charts show mean data  ± SEM from *n* = 4 experiments at 24 h for KG1a, NB4 and HL60, and at 72 h for KG1a and K562. **b** PBMCs from healthy donors were treated as shown for 24 h, stained with MitoSOX Red and carboxy-H_2_DCFDA and analysed by flow cytometry. Flow cytometry histograms show representative data; bar charts show mean ± SEM data from *n* = 4 donors. NB4, KG1a and HL60 cells were treated for 6 h before quantification of **c** reduced glutathione: oxidised glutathione (GSH:GSSG) ratios using the GSH/GSSG-Glo™ Assay (see ‘Methods’) and **d** Prostaglandin D2 (PGD2) by ELISA. Data are mean ± SEM for *n* = 4 experiments. Different letters indicate significant difference from other treatment groups (*p* < 0.05). Abbreviations: vehicle control (CON), 0.6 mM Valproic acid (VAL), 0.5 mM BEZ and 5 μM MPA (BaP 0.5 mM), 0.1 mM BEZ and 5 μM MPA (BaP 0.1 mM), the combination of Valproic acid 0.6 mM and BaP 0.1 mM (VBaP).

We have previously shown that AML cell killing by BaP 0.5 mM is directly related to the generation of reactive oxygen species (ROS) [11]. Thus it was important to investigate ROS production in the context of VBaP. As shown in Fig. 4a, BaP 0.5 mM treatment induced consistently higher ROS in HL60, KG1a, NB4 and K562 AML cells compared to all other treatments (Fig. 4a). However, VBaP consistently produced the next highest ROS production in all three AML cell lines at 24 h (Fig. 4a). VBaP also consistently induced greater ROS when measured at either 24 h or 72 h than either VAL or BaP 0.1 mM alone (Fig. 4a). In contrast to ROS, mitochondrial superoxide (mitosox) levels in NB4, KG1a and HL60 cells were largely unchanged in response to any of the treatments (Supplementary Fig. S2). Critically, no ROS or mitosox generation was seen in normal donor derived PBMCs in response to any of the treatments (Fig. 4b).

### VBaP induces oxidative stress in AMLs

During oxidative stress, the ratio of reduced glutathione (GSH) and oxidised glutathione (GSSG) levels decreases as cells attempt to moderate the damage caused by ROS [16]. We previously showed that ROS and prostaglandin production by BaP 0.5 mM resulted in depletion in cellular glutathione levels [11]. We, therefore, measured total glutathione and also GSH and GSSG individual levels (Supplementary Fig. S3) before and after treatments. Consistent with generation of ROS, BaP 0.5 mM, BaP 0.1 mM, VAL and VBaP reduced GSH levels with BaP 0.5 mM and VBaP having the most effect (Supplementary Fig. S3). GSH:GSSG ratios (Fig. 4c) were significantly reduced with all drug treatments addition of VAL to BaP 0.1 mM reducing the GSH:GSSG ratios to levels similar to that induced by BaP 0.5 mM in all AML cell lines tested.

### VBaP impacts lipid peroxidation and downstream prostaglandin synthesis in AML cells

Oxidative stress and ROS also activates the lipid peroxidation pathway [17]. We previously demonstrated that BaP 0.5 mM elevates lipid peroxidation in AML cells and that this leads to the downstream cyclooxygenase-independent generation and accumulation of PGD_2_ [11]. The rationale behind this observation was that BEZ driven ROS production caused lipid peroxidation and downstream generation of PGD_2_, and that simultaneously, MPA inhibits the metabolism of PGD_2_ by the aldoketoreductase AKR1C3 [18–20]. As shown in Supplementary Fig. 4A, BaP 0.5 mM again induced significant lipid peroxidation in all three cell lines tested. The reduction in lipid peroxidation was less with BaP 0.1 mM, and enhanced only marginally by the addition of Val (VBaP) in any of the lines (Supplementary Fig. S4A). These observations are consistent with and correlate with the relative generation of ROS by these treatments shown in Fig. 4a.

Analysis of PGD_2_ levels by ELISA highlighted variable basal levels between NB4, KG1a and HL60 cell lines (Fig. 4d). Consistent with its higher induction of ROS and lipid peroxidation, BaP 0.5 mM treatment led to the greatest increases in PGD_2_ production in all the lines (Fig. 4d). However, BaP 0.1 mM also significantly increased PGD_2_ production in all three cell lines which was significantly increased by the addition of VAL (Fig. 4d). VAL alone did not significantly increase PGD_2_ production in KG1a cells or HL60 cells, whereas a small increase was seen in NB4 cells (Fig. 4d). We have previously demonstrated that in AML cells the PGD_2_-11-ketoreductase activity of AKR1C3 metabolises PGD_2_ to form 11β-PGF_2_α. When not metabolised by AKR1C3, PGD_2_ undergoes a series of spontaneous dehydration reactions to form the anti-neoplastic and highly electrophilic derivative 15deoxy Δ^12,14^-PGJ_2_ (15d-PGJ_2_) [21, 22]. As observed for PGD_2_ levels, 15d-PGJ_2_ production in response to VBaP was significantly increased in NB4 and KG1a when compared to BaP 0.1 mM and again there was a trend towards an increase in HL60 cells (Supplementary Fig. S4B).

Thus, we demonstrate that the relative induction of ROS by our drug treatments coincide with glutathione depletion, lipid peroxidation and prostaglandin synthesis with addition of VAL to BaP 0.1 mM restoring levels closer to BaP 0.5 mM in all the cell lines tested.

### VBaP enhances ROS mediated damage by inhibiting Nrf2 function

**Fig. 5:**
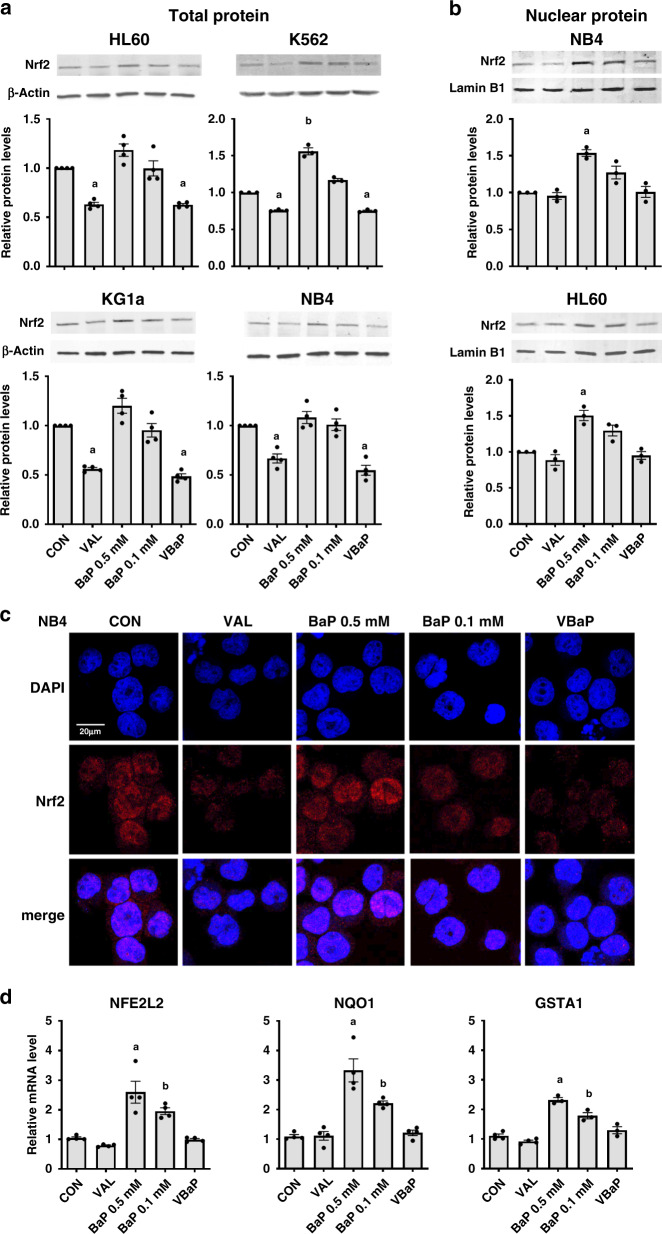
VBaP downregulates Nrf2 protein induction and activity. **a** HL60, K562, KG1a and NB4 cells were treated as shown for 4 h and protein levels of Nrf2 determined using western blotting using β-actin as a loading control. Panels show representative images of blots. Bar charts show total Nrf2 protein levels (normalised to β-actin) relative to control as mean ± SEM from *n* = 3 experiments. **b** NB4, and HL60 cells were treated as shown for 4 h and nuclear proteins extracted and immunoblotted for Nrf2 with Lamin B1 as a loading control. Panels show representative images of blots. Bar charts show relative nuclear Nrf2 protein levels (normalised to Lamin B levels) expressed as means ± SEM from *n* = 3 experiments. **c** NB4 cells were treated as shown for 4 h and cytospins prepared before fixing with paraformaldehyde and immunostaining staining for Nrf2 (red) and counterstaining nuclei with DAPI (blue). Scale bar indicates 20 μm. **d** NB4 cells were treated as shown for 4 h before extracting total RNA and synthesising cDNA. qRT-PCR was performed using gene-specific primers to Nrf2 target genes NFE2L2 (Nrf2), NAD(P)H dehydrogenase-quinone 1 (NQO1) and Glutathione S-transferase A1 (GSTA1). Data were normalised to 18s rRNA and calculated as relative mRNA compared to solvent controls. Bar charts show means ± SEM from *n* = 3 experiments. Different letters indicate significant difference from other treatment groups (*p* < 0.05). Abbreviations: vehicle control (CON), 0.6 mM Valproic acid (VAL), 0.5 mM BEZ and 5 μM MPA (BaP 0.5 mM), 0.1 mM BEZ and 5 μM MPA (BaP 0.1 mM), the combination of Valproic acid 0.6 mM and BaP 0.1 mM (VBaP).

Under conditions of oxidative stress, Nuclear factor erythroid 2-related factor 2 (Nrf2), the master transcriptional regulator of antioxidant response genes, translocates to the nucleus and activates its own expression as well as other key antioxidant genes. Consistent with ROS production, BaP 0.5 mM induced small increases in total Nrf2 levels in HL60, KG1a or NB4 cells and a significant increase in K562 cells (Fig. 5a). Despite inducing ROS, albeit at lower levels than BaP 0.5 mM, BaP 0.1 mM did not alter total Nrf2 protein levels. In marked contrast, VAL and VBaP exposure significantly reduced total Nrf2 protein levels in all four AML cell lines (Fig. 5a). Furthermore, western blots of nuclear protein extracts identified that, whereas both doses of BaP increased nuclear Nrf2 levels, VAL and VBaP did not significantly alter nuclear translocation of Nrf2 (Fig. 5b). This ability of VAL to suppress nuclear translocation of Nrf2 was confirmed using immunofluorescence staining of HL60 and NB4 cells. As shown in Fig. 5c for NB4, the addition of VAL alone reduced nuclear translocation of Nrf2 compared to untreated controls and similarly VBaP decreased nuclear localisation of Nrf2 compared to either BaP 0.5 mM or BaP 0.1 mM. Similar data were obtained for HL60 (Supplementary Fig. S5).

Finally, we used qRT-PCR to measure transcription levels of NFE2L2 which encodes for Nrf2, and the Nrf2 target genes NAD(P)H dehydrogenase [quinone] 1 (NQO1) and glutathione S-transferase A1 (GSTP1) [23]. Consistent with increased nuclear trafficking of Nrf2 and transcriptional activity, both BaP 0.5 mM and BaP 0.1 mM increased expression of NFE2L2 and GSTA1 mRNA in NB4, KG1a and HL60 cells (Fig. 5d and Supplementary Fig. S6). NQO1 mRNA levels were increased with BaP 0.5 mM in all three cell lines, and in BaP 0.1 mM treated NB4 and KG1a cells but not HL60 cells (Fig. 5d and Supplementary Fig. S4). The addition of VAL to BaP 0.1 mM attenuated this transcriptional response to oxidative stress in all three cell lines (Fig. 5d and Supplementary Fig. S6). Thus, these data indicate that VAL sensitises AML cells to BaP 0.1 mM by inhibiting Nrf2.

### Overexpression of Nrf2 abrogates the ability of VAL to enhance BAP 0.1 mM AML cell responses

**Fig. 6:**
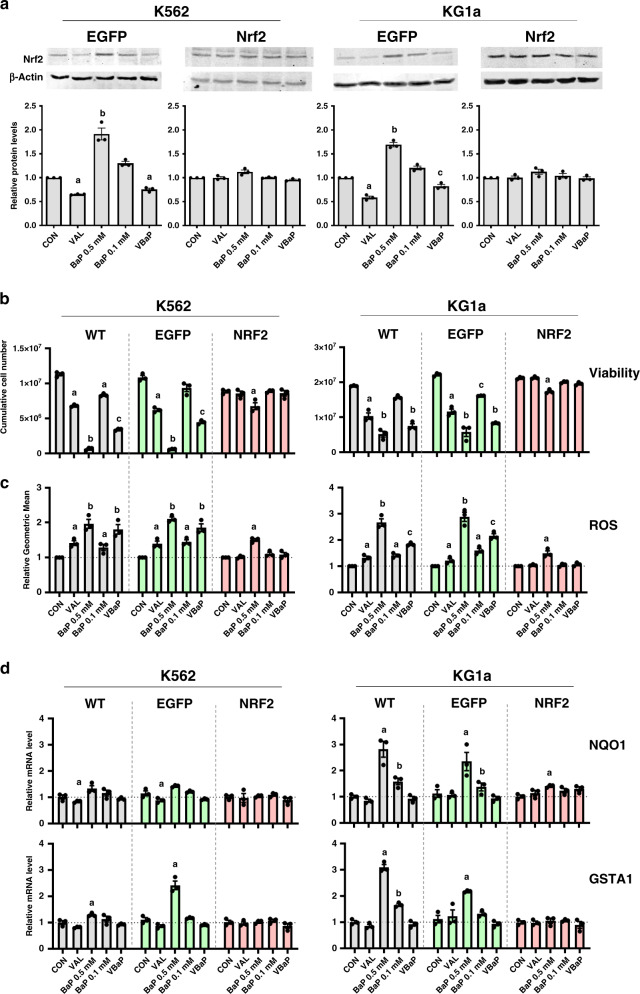
Nrf-2 overexpression reverses the ability of VAL to potentiate the effects of low dose BaP. **a** WT, control-EGFP and -NRF2 transfected K562 and KG1a cells were treated as shown for 4 h and protein levels of Nrf2 determined using western blotting using β-actin as a loading control. Panels show representative images of blots. Bar charts show total Nrf2 protein levels relative to control as mean ± SEM from *n* = 3 experiments. **b** WT, control-EGFP and -NRF2 transfected K562 and KG1a cells were treated as shown for 7 days, with feeding and retreating every 2 days, and numbers of live cells determined by flow cytometry using counting beads. Bar charts shows mean ± SEM for *n* = 3 experiments. **c** WT, control-EGFP and -NRF2 transfected K562 and KG1a cells were treated for 24 h, stained with carboxy-H_2_DCFDA for the detection of ROS and analysed by flow cytometry. Bar charts show mean data ± SEM from *n* = 3 experiments. **d** WT, control-EGFP and -NRF2 transfected K562 and KG1a cells were treated as shown for 4 h before extracting total RNA and synthesising cDNA. qRT-PCR was performed using gene-specific primers to Nrf2 target genes NAD(P)H dehydrogenase-quinone 1 (NQO1) and glutathione S-transferase A1 (GSTA1) expression. Data were normalised to 18s rRNA and calculated as relative mRNA levels compared to solvent controls. Bar graphs show mean ± SEM from *n* = 3 experiments. Different letters indicate significant difference from other treatment groups (*p* < 0.05). Abbreviations: vehicle control (CON), 0.6 mM Valproic acid (VAL), 0.5 mM BEZ and 5 μM MPA (BaP 0.5 mM), 0.1 mM BEZ and 5 μM MPA (BaP 0.1 mM), the combination of Valproic acid 0.6 mM and BaP 0.1 mM (VBaP).

In order to confirm the role of VAL-mediated suppression of Nrf2 responses in potentiating responses to BaP 0.1 mM, we constitutively expressed Nrf2 in K562 and KG1a AML. As previously observed for WT cells (Fig. 5a), Nrf2 protein expression was also reduced by VAL and VBaP compared to controls and BaP 0.1 mM in EGFP vector control transfectants of both K562 and KG1a (Fig. 6a). In contrast, Nrf2 levels did not reduce in Nrf2-overexpressing K562 and KG1a cells (Fig. 6a). As observed for wild type (WT) cells (Figs. 1a and 6b), VAL and VBaP enhanced killing of K562- and KG1a-EGFP empty vector control cells as compared to untreated controls and BaP 0.1 mM treated cells, respectively. In marked contrast, Nrf2 overexpression in KG1a and K562 cells completely rescued cells from VAL, BaP 0.1 mM and VBaP induced killing (Fig. 6b). Killing by BaP 0.5 mM was also almost completely rescued by overexpression of Nrf2 (Fig. 6b).

Similarly, as observed in WT cells (Figs. 4a and 6c), VAL and VBaP enhanced ROS production in EGFP empty vector control K562 and KG1a cells compared to untreated controls and BaP 0.1 mM treated cells respectively (Fig. 6c). In marked contrast, neither VAL nor VBaP induced ROS production in constitutively Nrf2-overexpressing K562 and KG1a cells and ROS induction by BaP 0.5 mM was significantly attenuated (Fig. 6a, b). Finally, constitutive expression of Nrf2 in KG1a cells also negated the capacity of VBaP to attenuate NQO1 and GSTA1 Nrf2-target genes compared to BAP 0.5 M treatment in either WT or EGFP empty vector control cells (Fig. 6d). Similarly, constitutive expression of Nrf2 in K562 cells negated the capacity of VBaP to attenuate GSTA1 Nrf2-target genes compared to BAP 0.5 mM in EGFP empty vector control cells. A similar, albeit statistically not significant, trend was observed for NQO1 expression in K562 cells.

## Discussion

This is a preclinical study addressing the failure to safely escalate BaP doses in AML patients in our most recent trial (ISRCTN99131400) compared to our first trial that showed efficacy and safety of continuous low dose BaP in elderly/relapsed AML (ISRCTN50635541) for sustained periods without complete remissions [10, 12]. We have shown here that the addition of clinically achievable doses of VAL to low dose BaP 0.1 mM (VBaP) recapitulates the killing of primary AML cells and AML cell lines seen in response to BaP 0.5 mM. Importantly, as expected for drugs that have been used safely in the clinic for decades, VBaP was tumour-selective with no loss in viability observed in normal donor PBMCs and bone marrow-derived HSPCs.

Whilst elucidating the mechanism of action of BaP in earlier studies, we had previously identified that BaP and BEZ suppressed de novo fatty acid synthesis by downregulating SCD1, a key rate-limiting enzyme involved in synthesising palmitoleic acid (16:1) and oleic acid (18:1), essential precursors for intracellular free fatty acid synthesis. As expected, BaP 0.1 mM had less effect than BaP 0.5 mM in lowering SCD1 levels, however, the addition of VAL was able to restore this activity (Fig. 3). Reductions in SCD1 were also reflected in similar patterns in changes in 16:1 and 18:1 fatty acid levels with VBaP. In another study, changes in fatty acid pathways were also identified by untargeted serum metabolomic profiling in 44 AML patients receiving ATRA and VAL combined with low-dose cytotoxic drugs (cytarabine, hydroxyurea, 6-mercaptopurin) [24]. Twenty-three metabolites, the majority associated with fatty acid metabolism and amino acid pathways, were significantly altered by seven-day valproic acid treatment [24]. VAL has also been demonstrated to alter key genes involved in adipogenesis, including reductions in SCD1 mRNA and protein expression, in 3T3-L1 cells induced to differentiate into adipocytes [25].

ROS are important regulators of cellular signalling in both physiological and pathological cellular processes and are elevated in many cancers including AML [14, 26, 27] and MDS [28, 29]. The cytotoxicity of many anti-AML therapies, e.g. cytarabine and daunorubicin in AML [30] is mediated at least in part by induction of ROS. Nrf2 is a master regulator of oxidative stress regulating expression of genes involved in neutralising ROS and aberrant Nrf2 activity has been shown to contribute to development, progression and chemotherapy resistance in multiple cancers including AML [31, 32]. VAL has been shown to suppress Nrf2 expression in multiple cancers, sensitising them to oxidative stress [33–35]. We demonstrate here that this is also the case in AML cells. We have previously shown that oxidative stress is induced in a dose-dependent manner by BEZ in BaP and ROS levels correlate with killing in AML cells [11]. Hence, whilst BaP 0.1 mM induces lower levels of ROS than BaP 0.5 mM, attenuation of the Nrf2 response by VAL in VBaP restores the cellular effects of BaP 0.1 mM to almost BaP 0.5 mM levels including lipid peroxidation, GSH:GSSG levels and ratios and PGD_2_ and 15dPGJ_2_ levels. Importantly, whilst AML cells were highly susceptible to BaP and VBaP induced ROS, normal donor PBMCs and HSPCs were resistant. Hence, VBaP induces oxidative stress in a tumour-selective manner.

Several trials have investigated VAL as adjunctive therapy in AML alongside ATRA, decitabine, theophylline or azacytidine [11, 36–38]. These trials used VAL at doses higher than typically used in the management of epilepsy. At these high doses, VAL exhibits histone deacetylase activity but is also associated with adverse events in AML patients [39, 40]. In vitro studies experiments have demonstrated variable dose-dependent responses to VAL [41, 42]. Here, we have demonstrated an alternative mode of action for VAL in AML cells that can be exploited at lower VAL doses. Whilst, there are many factors influencing the clinical suitability and pharmacokinetics of VAL, serum concentrations of VAL consistent with our study are achievable in elderly patients using well-tolerated doses [43, 44] and we propose that clinical studies of VBaP are relevant and warranted.

A major challenge in our previous BaP trials has been the focus on elderly, relapsed/refractory AML patients for whom more intensive therapies were not option. Prognosis for these types of patients is very poor with a median survival of 7 weeks and consistent with this, 5/20 patients died within four weeks of commencing therapy. Despite this, we still managed to achieve sustained haematological responses using low dose BaP [10]. Therefore, we would propose that treating patients earlier in their disease progression with low toxicity therapies is the most effective clinical strategy for improving patient outcomes.

Approximately 10% of AML arises from transformation of MDS. Like AML, MDS are a heterogeneous group of myeloid neoplasms with diverse clinical courses. For many of these patients there are very few treatment options other than regular transfusions to combat life-threatening deficits in red cells and platelets and antibiotic control of frequent life threatening infections. It is therefore an attractive option to consider testing VBaP in MDS patients. The episodes of improved erythrocyte, platelet and neutrophil counts and diminished transfusion dependency observed in AML patients in our first trial [10] would represent real clinical benefit in MDS patients. In addition, the anti-AML activity of VBaP demonstrated here may delay and/or reduce transformation rates to AML. Thus, VBaP may have profound impact on quality of life and progression free survival in MDS patients. To test this, we have obtained funding and ethical approval for Repair-MDS, a phase 2 clinical trial comparing VBaP versus danazol [45] in MDS patients. This study will assess the impact of VBaP versus danazol on transfusion dependence, quality of life and transformation to AML.

Importantly, there is increasing evidence that the NRF2 pathway is a driver of progression, metastasis, and resistance to therapy in many cancers [46]. Therefore, our findings demonstrating suppression of NRF2 activity by VAL highlight the potential of VAL as potential adjunctive therapy in other cancers.

## Supplementary information

Reproducibility Checklist

Supplementary Data

## Data Availability

All relevant data is available in the manuscript.
