# Supplementary material for: Valproic acid disables the Nrf2 anti-oxidant response in acute myeloid leukaemia cells enhancing reactive oxygen species-mediated killing
Source: Br J Cancer. 2021 Oct 22;126(2):275–86. doi: 10.1038/s41416-021-01570-z (PMC8770569; doi:10.1038/s41416-021-01570-z)
Supplement: Supplementary file 2 — Supplementary Data [file 41416_2021_1570_MOESM2_ESM.pptx]

## Slide 1
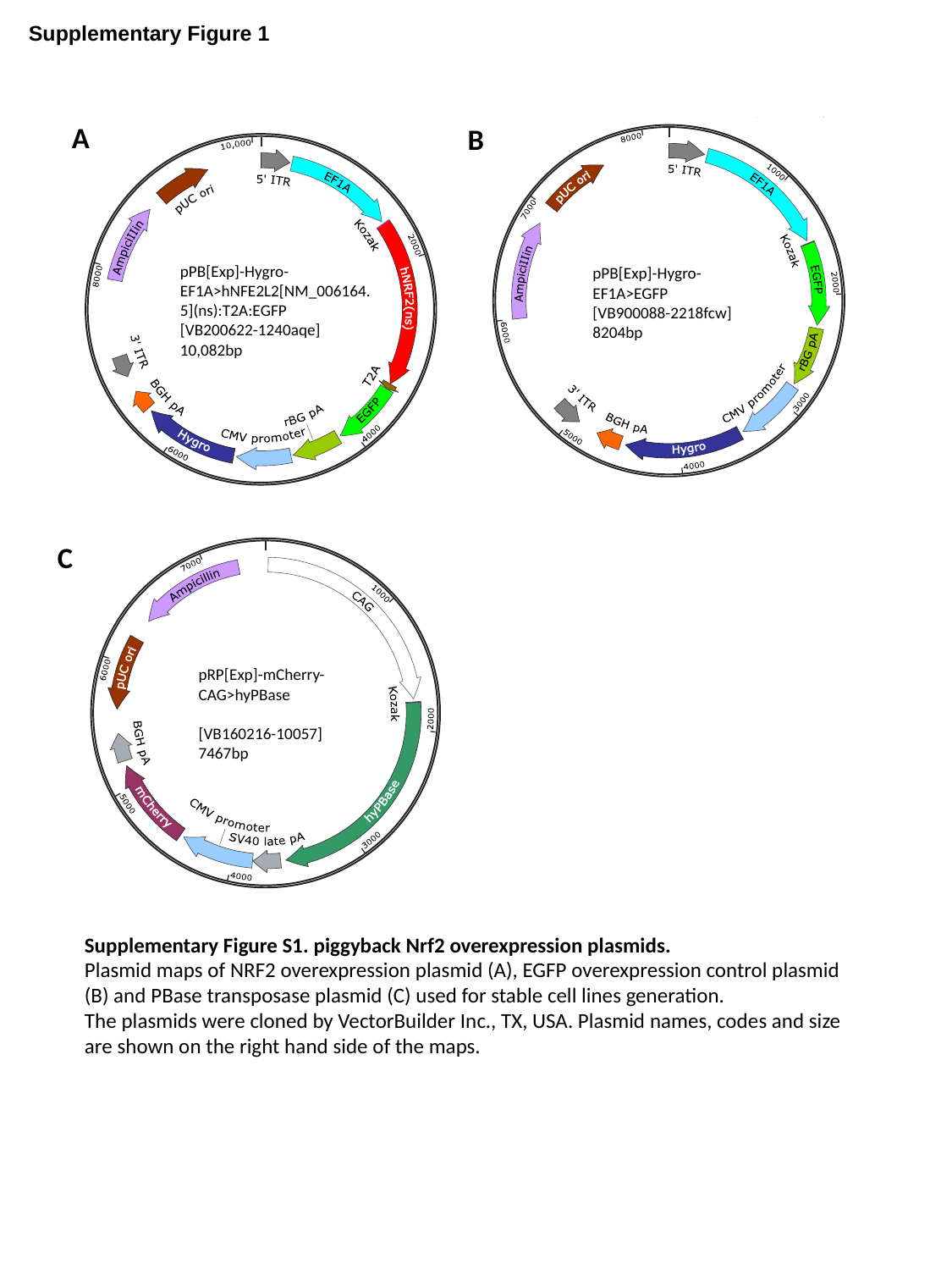

Supplementary Figure 1
A
B
pPB[Exp]-Hygro-EF1A>EGFP
[VB900088-2218fcw]
8204bp
pPB[Exp]-Hygro-EF1A>hNFE2L2[NM_006164.5](ns):T2A:EGFP
[VB200622-1240aqe]
10,082bp
C
pRP[Exp]-mCherry-CAG>hyPBase
[VB160216-10057]
7467bp
Supplementary Figure S1. piggyback Nrf2 overexpression plasmids.
Plasmid maps of NRF2 overexpression plasmid (A), EGFP overexpression control plasmid (B) and PBase transposase plasmid (C) used for stable cell lines generation.
The plasmids were cloned by VectorBuilder Inc., TX, USA. Plasmid names, codes and size are shown on the right hand side of the maps.

## Slide 2
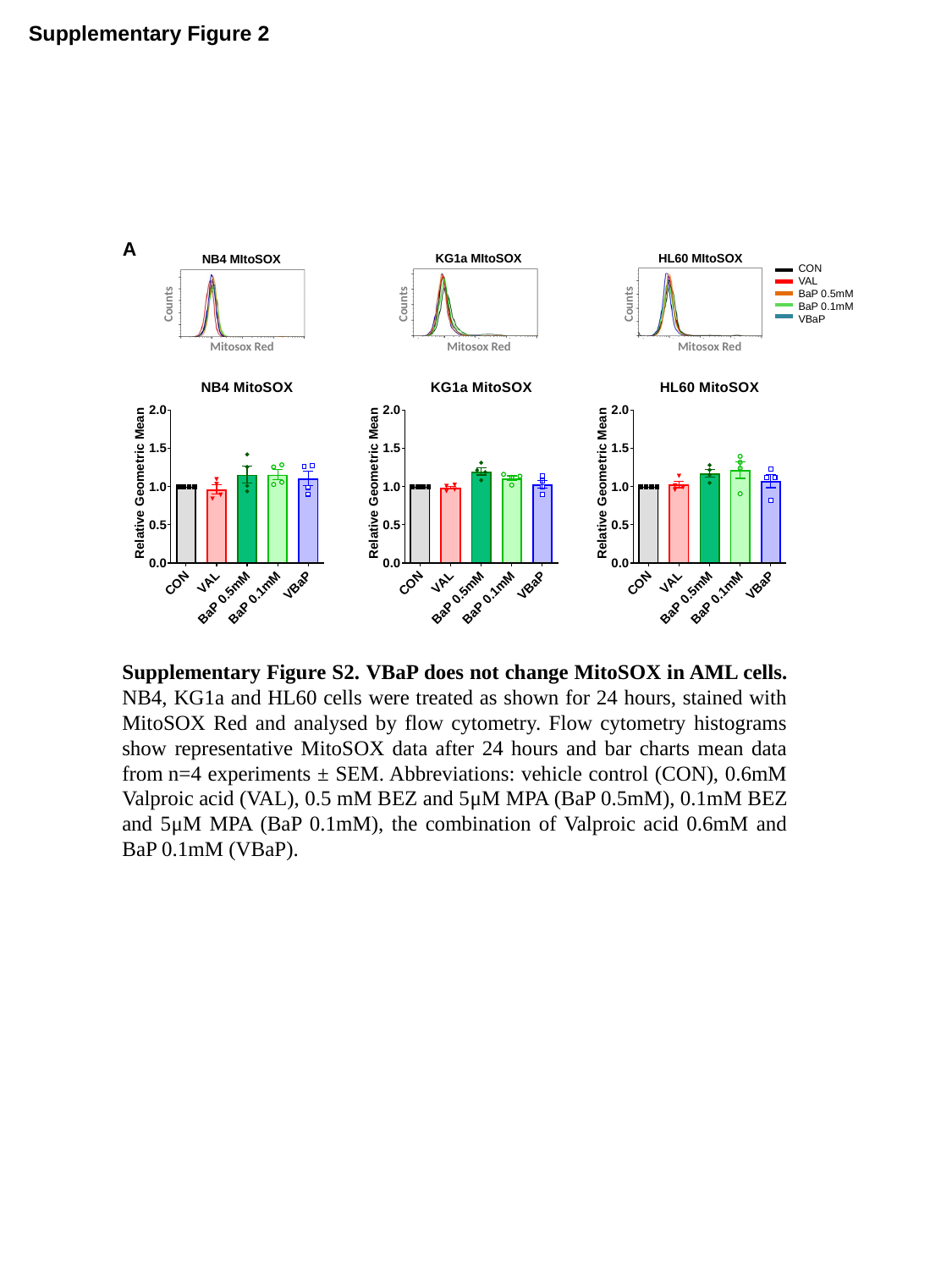

Supplementary Figure 2
A
CON
VAL
BaP 0.5mM BaP 0.1mM VBaP
KG1a MItoSOX
HL60 MItoSOX
NB4 MItoSOX
Counts
Counts
Counts
Mitosox Red
Mitosox Red
Mitosox Red
Supplementary Figure S2. VBaP does not change MitoSOX in AML cells. NB4, KG1a and HL60 cells were treated as shown for 24 hours, stained with MitoSOX Red and analysed by flow cytometry. Flow cytometry histograms show representative MitoSOX data after 24 hours and bar charts mean data from n=4 experiments ± SEM. Abbreviations: vehicle control (CON), 0.6mM Valproic acid (VAL), 0.5 mM BEZ and 5μM MPA (BaP 0.5mM), 0.1mM BEZ and 5μM MPA (BaP 0.1mM), the combination of Valproic acid 0.6mM and BaP 0.1mM (VBaP).

## Slide 3
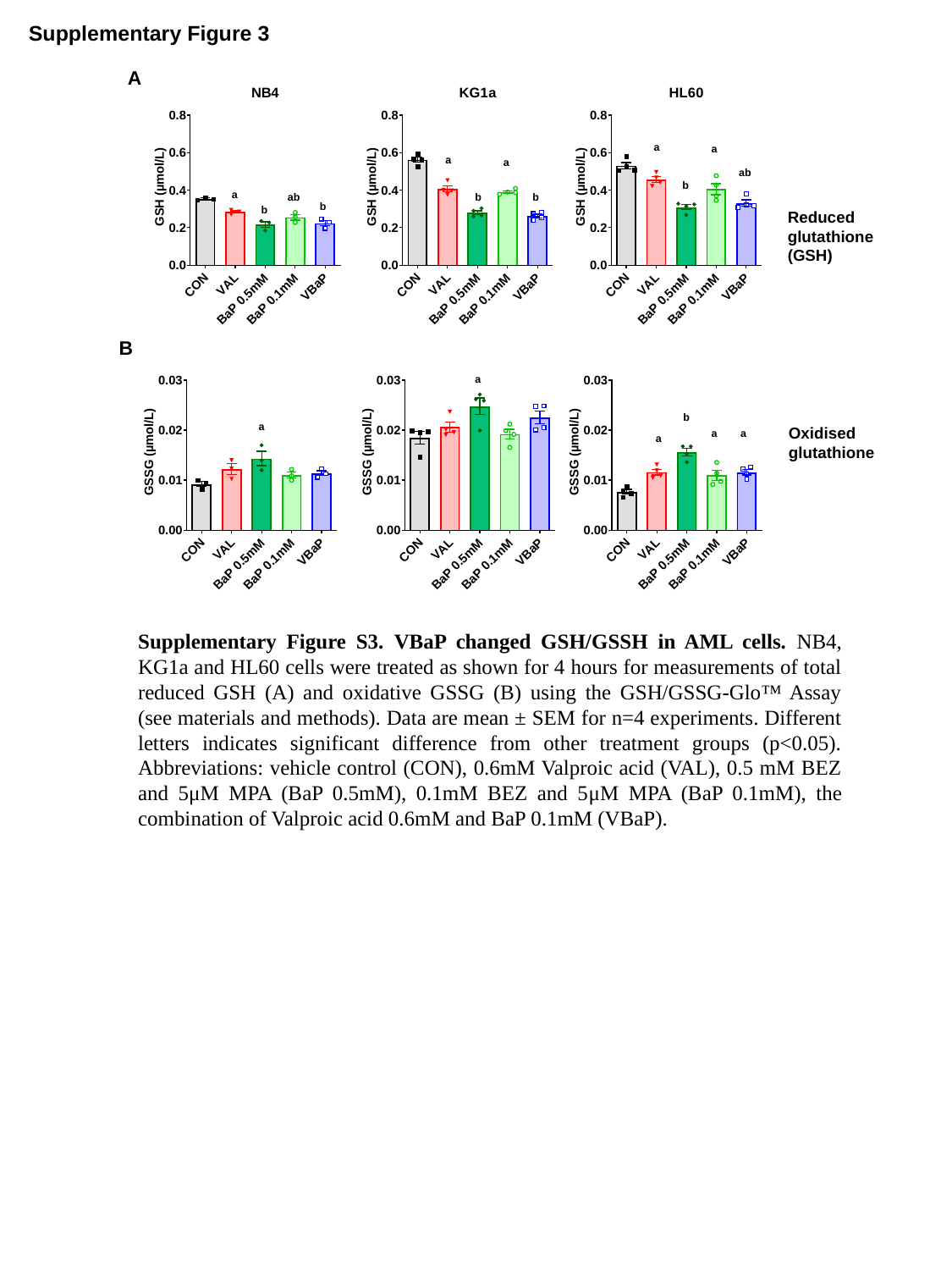

Supplementary Figure 3
A
a
a
a
a
ab
b
a
ab
b
b
b
Reduced glutathione
(GSH)
b
B
a
b
Oxidised glutathione
a
a
a
a
Supplementary Figure S3. VBaP changed GSH/GSSH in AML cells. NB4, KG1a and HL60 cells were treated as shown for 4 hours for measurements of total reduced GSH (A) and oxidative GSSG (B) using the GSH/GSSG-Glo™ Assay (see materials and methods). Data are mean ± SEM for n=4 experiments. Different letters indicates significant difference from other treatment groups (p<0.05). Abbreviations: vehicle control (CON), 0.6mM Valproic acid (VAL), 0.5 mM BEZ and 5μM MPA (BaP 0.5mM), 0.1mM BEZ and 5μM MPA (BaP 0.1mM), the combination of Valproic acid 0.6mM and BaP 0.1mM (VBaP).

## Slide 4
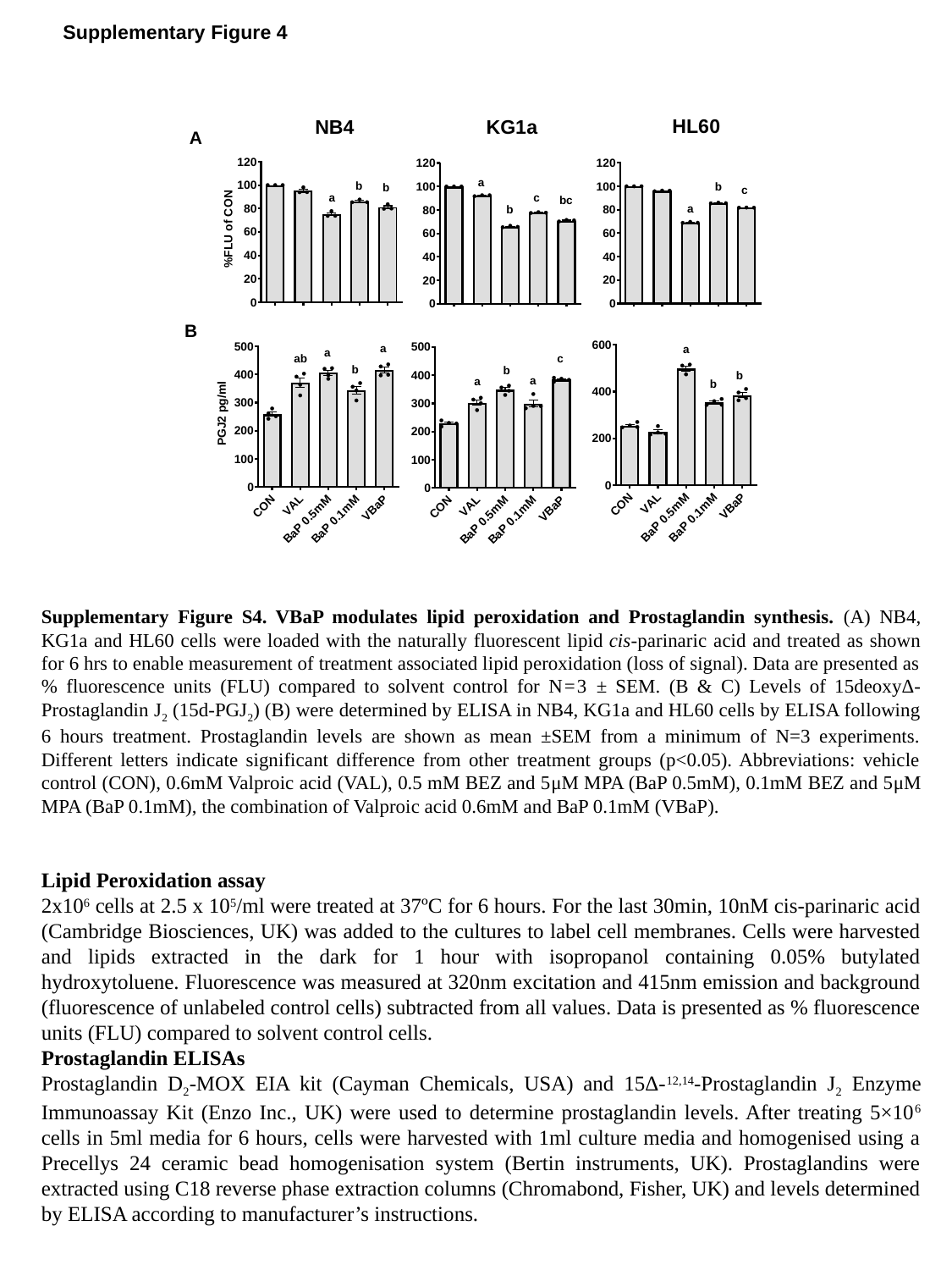

Supplementary Figure 4
HL60
NB4
KG1a
A
a
b
b
b
c
a
c
bc
a
b
B
a
a
a
ab
c
b
b
b
a
a
b
Supplementary Figure S4. VBaP modulates lipid peroxidation and Prostaglandin synthesis. (A) NB4, KG1a and HL60 cells were loaded with the naturally fluorescent lipid cis-parinaric acid and treated as shown for 6 hrs to enable measurement of treatment associated lipid peroxidation (loss of signal). Data are presented as % fluorescence units (FLU) compared to solvent control for N = 3 ± SEM. (B & C) Levels of 15deoxyΔ-Prostaglandin J2 (15d-PGJ2) (B) were determined by ELISA in NB4, KG1a and HL60 cells by ELISA following 6 hours treatment. Prostaglandin levels are shown as mean ±SEM from a minimum of N=3 experiments. Different letters indicate significant difference from other treatment groups (p<0.05). Abbreviations: vehicle control (CON), 0.6mM Valproic acid (VAL), 0.5 mM BEZ and 5μM MPA (BaP 0.5mM), 0.1mM BEZ and 5μM MPA (BaP 0.1mM), the combination of Valproic acid 0.6mM and BaP 0.1mM (VBaP).
Lipid Peroxidation assay
2x10^6^ cells at 2.5 x 10^5^/ml were treated at 37ºC for 6 hours. For the last 30min, 10nM cis-parinaric acid (Cambridge Biosciences, UK) was added to the cultures to label cell membranes. Cells were harvested and lipids extracted in the dark for 1 hour with isopropanol containing 0.05% butylated hydroxytoluene. Fluorescence was measured at 320nm excitation and 415nm emission and background (fluorescence of unlabeled control cells) subtracted from all values. Data is presented as % fluorescence units (FLU) compared to solvent control cells.
Prostaglandin ELISAs
Prostaglandin D2-MOX EIA kit (Cayman Chemicals, USA) and 15Δ-12,14-Prostaglandin J2 Enzyme Immunoassay Kit (Enzo Inc., UK) were used to determine prostaglandin levels. After treating 5×10^6^ cells in 5ml media for 6 hours, cells were harvested with 1ml culture media and homogenised using a Precellys 24 ceramic bead homogenisation system (Bertin instruments, UK). Prostaglandins were extracted using C18 reverse phase extraction columns (Chromabond, Fisher, UK) and levels determined by ELISA according to manufacturer’s instructions.

## Slide 5
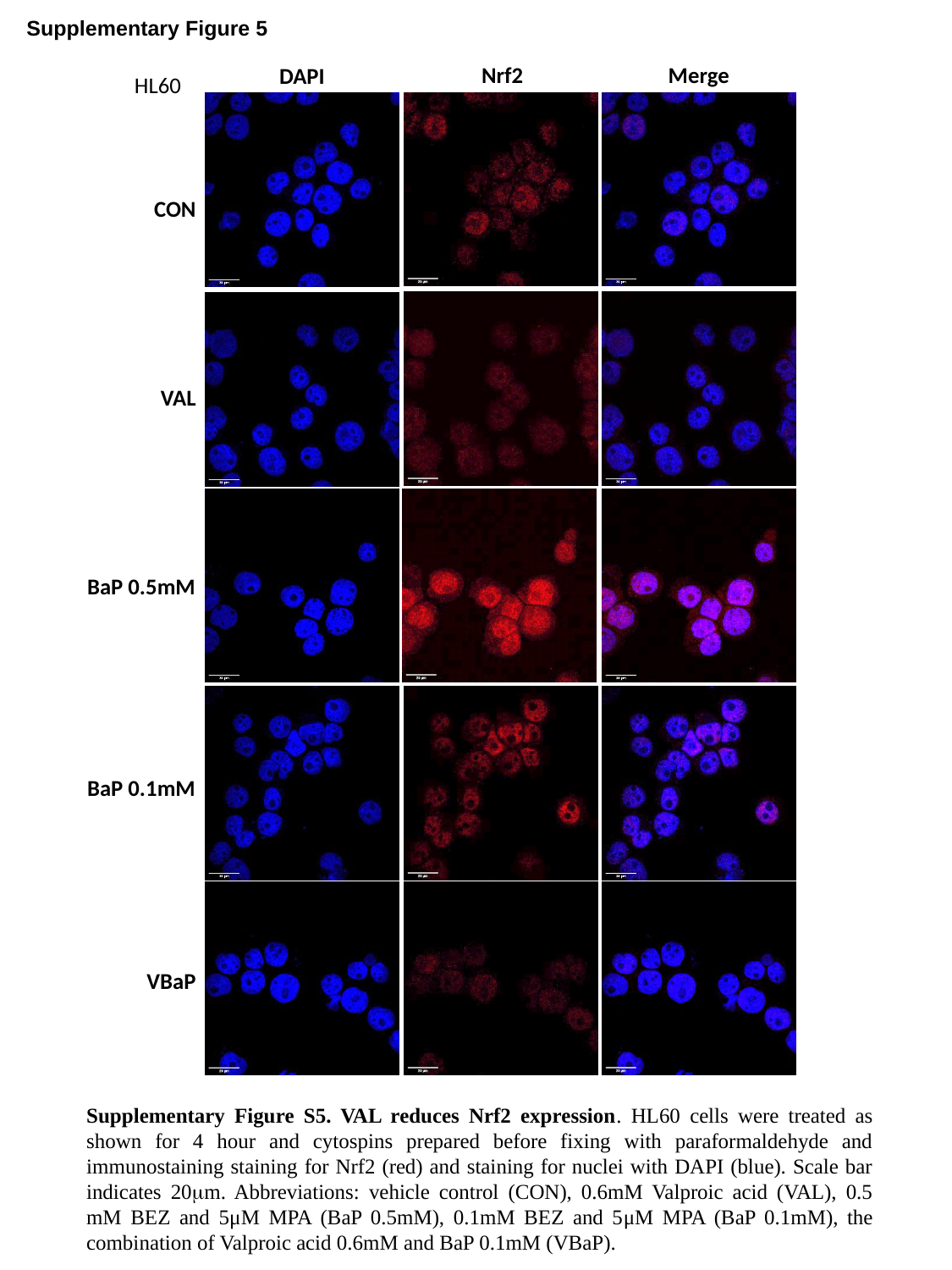

Supplementary Figure 5
Merge
Nrf2
DAPI
HL60
CON
VAL
BaP 0.5mM
BaP 0.1mM
VBaP
Supplementary Figure S5. VAL reduces Nrf2 expression. HL60 cells were treated as shown for 4 hour and cytospins prepared before fixing with paraformaldehyde and immunostaining staining for Nrf2 (red) and staining for nuclei with DAPI (blue). Scale bar indicates 20mm. Abbreviations: vehicle control (CON), 0.6mM Valproic acid (VAL), 0.5 mM BEZ and 5μM MPA (BaP 0.5mM), 0.1mM BEZ and 5μM MPA (BaP 0.1mM), the combination of Valproic acid 0.6mM and BaP 0.1mM (VBaP).

## Slide 6
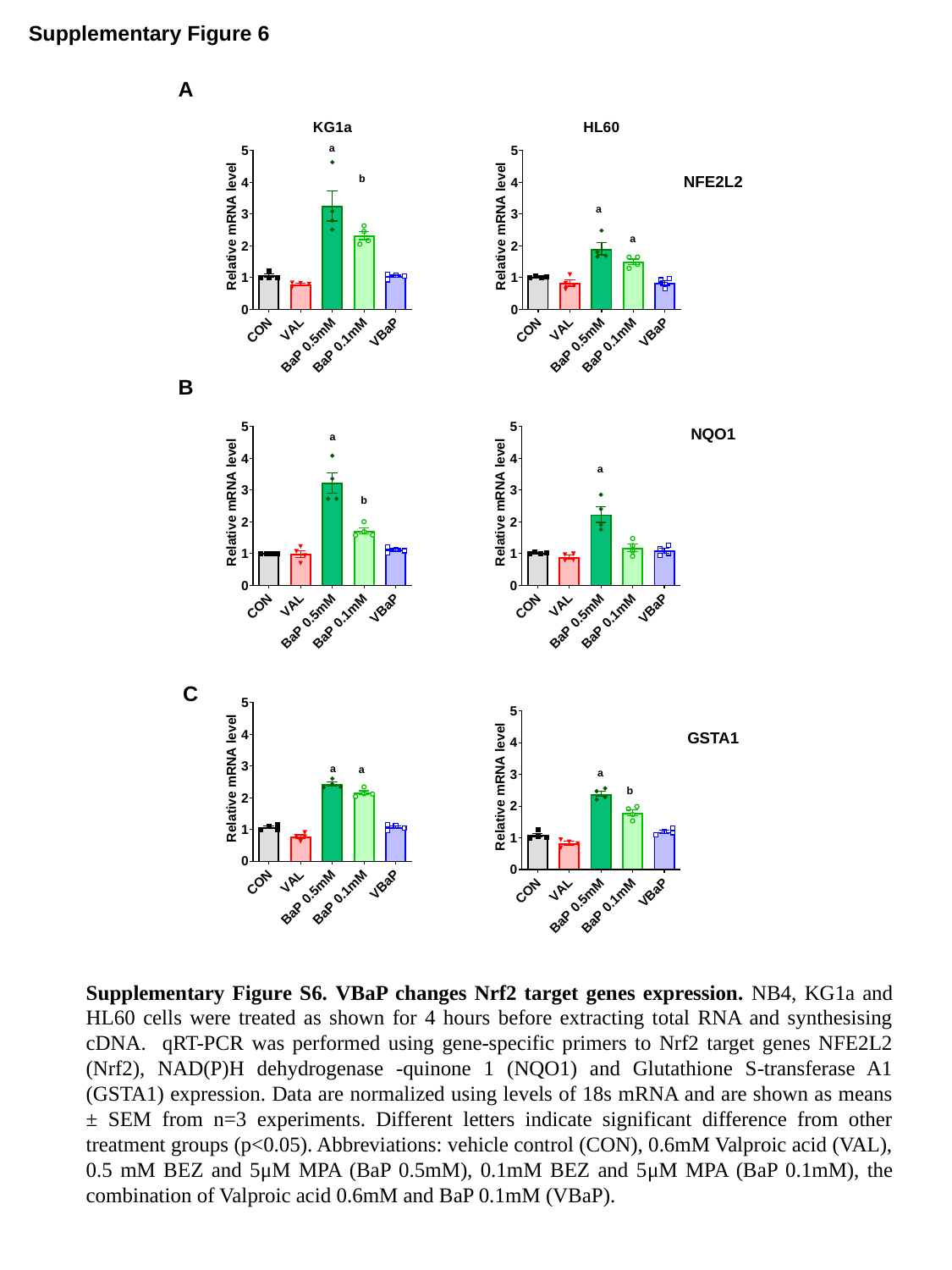

Supplementary Figure 6
A
a
NFE2L2
b
a
a
B
NQO1
a
a
b
C
GSTA1
a
a
a
b
Supplementary Figure S6. VBaP changes Nrf2 target genes expression. NB4, KG1a and HL60 cells were treated as shown for 4 hours before extracting total RNA and synthesising cDNA. qRT-PCR was performed using gene-specific primers to Nrf2 target genes NFE2L2 (Nrf2), NAD(P)H dehydrogenase -quinone 1 (NQO1) and Glutathione S-transferase A1 (GSTA1) expression. Data are normalized using levels of 18s mRNA and are shown as means ± SEM from n=3 experiments. Different letters indicate significant difference from other treatment groups (p<0.05). Abbreviations: vehicle control (CON), 0.6mM Valproic acid (VAL), 0.5 mM BEZ and 5μM MPA (BaP 0.5mM), 0.1mM BEZ and 5μM MPA (BaP 0.1mM), the combination of Valproic acid 0.6mM and BaP 0.1mM (VBaP).
